# Supplementary material for: Comparative multiomics analysis of cell physiological state after culture in a basket bioreactor
Source: Sci Rep. 2022 Nov 23;12:20161. doi: 10.1038/s41598-022-24687-4 (PMC9686226; doi:10.1038/s41598-022-24687-4)
Supplement: Supplementary file 1 — Supplementary Information 1. [file 41598_2022_24687_MOESM1_ESM.zip › raw data/Transcriptomic raw data/6.Enrichment/2.KEGG/TE1vsTC1/ALL/src/csab03018.html]

csab03018


- csab:103218486

- Down regulated genes

103218486(-1.5200283537911)

- csab:103234733

- Up regulated genes

103234733(1.15751068757509)

- csab:103242916

- Up regulated genes

103242916(1.272363135682)

- csab:103218486

- Down regulated genes

103218486(-1.5200283537911)

Close
